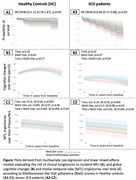# Association between mediterranean‐like diet, longitudinal cognitive decline, and medial temporal atrophy over time in cognitively unimpaired older adults with or without SCD

**DOI:** 10.1002/alz.090659

**Published:** 2025-01-03

**Authors:** Elizabeth Kuhn, Debora Melo van Lent, Melina Stark, Luca Kleineidam, Renat Yakupov, Oliver Peters, Josef Priller, Anja Schneider, Klaus Fliessbach, Jens Wiltfang, Emrah Düzel, Katharina Buerger, Robert Perneczky, Stefan Teipel, Christoph Laske, Annika Spottke, Frank Jessen, Michael Wagner, for the DELCODE study group

**Affiliations:** ^1^ German Center for Neurodegenerative Diseases (DZNE), Bonn Germany; ^2^ Department of Neurodegenerative Diseases and Geriatric Psychiatry, University of Bonn Medical Center, Bonn Germany; ^3^ Glenn Biggs Institute for Alzheimer’s & Neurodegenerative Diseases, University of Texas Health San Antonio, San Antonio, TX USA; ^4^ German Center for Neurodegenerative Diseases (DZNE), Magdeburg Germany; ^5^ Institute of Cognitive Neurology and Dementia Research (IKND), Otto‐von‐Guericke University, Magdeburg Germany; ^6^ Charité – Universitätsmedizin Berlin, corporate member of Freie Universität Berlin and Humboldt‐Universität zu Berlin – Institute of Psychiatry and Psychotherapy, Berlin Germany; ^7^ German Center for Neurodegenerative Diseases (DZNE), Berlin Germany; ^8^ School of Medicine, Technical University of Munich; Department of Psychiatry and Psychotherapy, Munich Germany; ^9^ University of Edinburgh and UK DRI, Edinburgh United Kingdom; ^10^ Department of Psychiatry and Psychotherapy, Charité, Charitéplatz 1, Berlin Germany; ^11^ Department of Psychiatry and Psychotherapy, University Medical Center, University of Goettingen, Goettingen Germany; ^12^ German Center for Neurodegenerative Diseases (DZNE), Goettingen Germany; ^13^ Neurosciences and Signaling Group, Institute of Biomedicine (iBiMED), Department of Medical Sciences, University of Aveiro, Aveiro Portugal; ^14^ Institute for Stroke and Dementia Research (ISD), University Hospital, LMU, Munich Germany; ^15^ German Center for Neurodegenerative Diseases (DZNE), Munich Germany; ^16^ LMU University Hospital, Munich Germany; ^17^ Munich Cluster for Systems Neurology (SyNergy), Munich Germany; ^18^ Department of Psychiatry and Psychotherapy, University Hospital, LMU Munich, Munich Germany; ^19^ German Center for Neurodegenerative Diseases, Rostock Germany; ^20^ Department of Psychosomatic Medicine, Rostock University Medical Center, Rostock Germany; ^21^ German Center for Neurodegenerative Diseases (DZNE), Tübingen Germany; ^22^ Section for Dementia Research, Hertie Institute for Clinical Brain Research and Department of Psychiatry and Psychotherapy, University of Tuebingen, Tuebingen Germany; ^23^ Department of Neurology, University of Bonn, Bonn Germany; ^24^ Department of Psychiatry, University of Cologne, Medical Faculty, Cologne Germany; ^25^ Excellence Cluster on Cellular Stress Responses in Aging‐Associated Diseases (CECAD), University of Cologne, Cologne Germany

## Abstract

**Background:**

Previous findings evaluating longitudinal cognition in relation to the MeDi diet are inconsistent, and few studies have examined it in relation to the presence/absence of subjective cognitive decline (SCD). Our current aims are to test whether adherence to the MeDi diet is associated with the risk of clinical progression, future cognitive decline, and atrophy over time in Alzheimer’s disease (AD)‐sensitive regions in cognitively unimpaired (CU) older adults with or without SCD.

**Methods:**

This longitudinal study includes 171 controls and 228 SCD patients recruited from memory clinics in the DELCODE study. All participants underwent serial neuropsychological assessments and MRI scans for up to 7 years, and had available MeDi adherence scores based on the clustered Food Frequency Questionnaire (range: 0‐9). Clinical progression to incident‐MCI status was determined by consensus diagnosis, global cognitive decline was assessed using the PACC5 cognitive composite, and medial temporal lobe (MTL) gray matter volume was estimated using the Freesurfer longitudinal pipeline. We used multivariate Cox regression and linear mixed‐effects modeling to address our objectives. Gray matter volumes were corrected for total intracranial volume, and all analyses were adjusted for demographics.

**Results:**

In SCD patients, we found that higher adherence to the MeDi diet was associated with a lower risk of clinical progression to incident‐MCI (**Fig. A2**), and less global cognitive decline (**Fig. B2**), and MTL atrophy over time (**Fig. C2;** mainly driven by less parahippocampal and amygdala atrophy). Higher adherence to the MeDi diet was also associated with better baseline cognition and MTL volumes in these patients. This was not significant in the HC group (**Fig. A1‐C1**), possibly because they experienced less cognitive decline over time than the SCD group (both p<0.001; not significant for MTL atrophy). Further adjustment for kcal intake, body mass index, and physical activity yielded similar results, although sometimes only at trend levels.

**Conclusion:**

In SCD patients seeking medical help, better adherence to a MeDi diet pattern may reduce future cognitive decline and MTL atrophy. These novel data provide a rationale for dietary intervention studies in this population, and support counseling SCD patients on the benefits of MeDi (and other lifestyle factors) for cognitive health.